# Nutritional Composition and Acceptability of Egg Powder‐Fortified Tom Brown Among School‐Aged Children in the Wa Municipality

**DOI:** 10.1002/fsn3.70288

**Published:** 2025-07-14

**Authors:** Joe Dare Nyefene, Felix Mills‐Robertson, Isaac Amoah, Charles Apprey, Christopher Edeh

**Affiliations:** ^1^ Department of Biochemistry and Biotechnology, College of Science Kwame Nkrumah University of Science and Technology Kumasi Ghana; ^2^ Department of Hotel, Catering and Institutional Management Dr. Hilla Limann Technical University Wa Ghana; ^3^ Emmppek Farms Ltd Warri Nigeria

**Keywords:** composite product, egg powder‐fortified, fortification, malnutrition, phytate, therapeutic diet

## Abstract

The study developed a nutrient‐rich composite food by blending roasted corn flour with egg powder, targeting school‐aged children to address protein‐energy and micronutrient deficiencies. The objective of the study was to evaluate the functional properties (viscosity, solubility), acceptability, and shelf life stability of egg powder‐fortified staples and provide an understanding of how nutrient fortification ratios such as egg powder: corn flour affect sensory preferences and compliance in study populations, especially children. The composite significantly enhanced protein, carbohydrates, iron, zinc, magnesium, folate, and vitamin A compared to plain corn flour, supporting growth, immunity, and cognitive health and contributing 573 kcal per 250 mL serving, translating into about 34.7% of the average RDA of energy for children 5–12 years old. Consuming 250 mL of the egg powder‐fortified Tom Brown contributes 6.66 mg of iron, representing about 74% of the 9 mg RDA of iron for children within 8–12 and 17.34 g of protein. Functional tests showed favorable bulk density (0.65 g/mL), solubility (23%), and a 12‐month shelf life, ensuring practicality in resource‐limited settings. Sensory evaluations of appearance, aroma, taste, texture, and general acceptability with 75 participants and samples randomly presented to the pupils and the randomization order generated using the Compusense Cloud software ranked the 100:20 corn‐to‐egg ratio highest in acceptability. The formulation offers a scalable, culturally appropriate solution to improve hemoglobin levels, reduce anemia, and boost nutritional resilience, positioning it as a transformative tool in combating global childhood malnutrition.

## Graphical abstract

The study evaluated nutritional and functional properties of corn flour‐egg powder composite to create a nutrient‐dense meal for children. The composite had enhanced protein (18.09%) and carbohydrates (61.99%), with improved iron, zinc, magnesium, vitamin A, and folate levels. Functional properties showed good viscosity (168 RVU) and stability (12‐month shelf life). Sensory evaluation favored the 100:20 egg powder‐to‐corn flour ratio for taste and acceptability. These findings highlight its potential as a fortified intervention food to improve children's nutrition and hemoglobin levels.

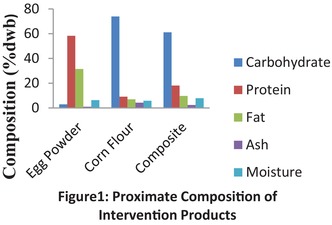

## 1 Introduction

Malnutrition remains a significant public health challenge in many developing countries, including Ghana, taking various forms such as protein‐energy malnutrition and deficiencies in essential vitamins and minerals (Adu‐Afarwuah et al. 2023). These nutritional gaps adversely affect children's cognitive development, growth, and overall health (Bommer et al. 2020). Therefore, a nutrient‐rich diet is crucial for children's rapid development.

Traditional weaning foods such as Tom Brown, a porridge made from roasted maize, millet, legumes, and other grains, are common in Ghanaian diets. Tom Brown is valued for its availability and popularity across communities. It has been effectively used as a supplementary therapeutic diet for treating moderate acute malnutrition, demonstrating good recovery and low mortality rates (Sewor and Jayalakshmi 2024). Research indicates that adding hydrothermally treated soybeans or biofortified maize to Tom Brown improves its nutritional value and acceptance (Kalumbi et al. 2019; Siwela et al. 2020).

Despite its benefits, Tom Brown does not contain all the key nutrients that are essential for optimal child growth. Many Ghanaian children, especially in rural areas, do not receive the necessary nutrients, leading to widespread stunting and undernutrition (Aryeetey et al. 2022). A study on complementary food mixes in Ghana linked unfortified plant‐based foods to prevalent infant malnutrition (Sewor and Jayalakshmi 2024). Also, a study on how dietary diversity is related to the hematological status of preschool children in Ghana revealed that anemia prevalence (Hb < 11 g/dL) among children aged 6–59 months was 66.8% (Saaka and Galaa 2017). In a similar study to determine anemia prevalence and its predictors among children under five years in Ghana using a multilevel analysis of the Cross‐sectional 2019 Ghana Malaria Indicator Survey and cited in (Aheto et al. 2023) indicated that 54% of children under 5 years were anemic. Additionally, poor complementary feeding practices exacerbate the issue, with 14% of infants introduced to these foods before three months of age (Wiafe et al. 2023).

Though popular, conventional cereal and legume mixes do not meet recommended nutrient levels for certain nutrients, especially the micronutrients and fats (Temba et al. 2016). Enhancing the diet with grain legumes can improve protein and micronutrient adequacy (De Jager et al. 2019). Consequently, with its proven efficacy in treating mild acute malnutrition, *Tom Brown* can be a vital nutritional source for children. Roasting grains improves digestibility and reduces antinutritional factors such as phytates and tannins, further aiding nutrient absorption (Mahanta et al. 2020).

On the other hand, eggs are a rich source of high‐quality protein, essential vitamins, and minerals, making them crucial for human nutrition (Rafed et al. 2024). Nutrients such as riboflavin, selenium, choline, and vitamins A, D, B_12_, and B_9_ are abundant in eggs. Egg powder retains these nutritional benefits while at the same time offering a longer shelf life and straightforward incorporation into various dishes (Wulandari and Arief 2022). Besides, iron from egg powder is more bioavailable than legume sources. Egg powder fortification can significantly enhance the nutritional profile of Tom Brown, addressing its deficiencies and boosting its overall health benefits. This is particularly valuable in resource‐limited settings where eggs are readily available due largely to some extent domestic rearing of fowls and the relative affordability (Javed et al. 2018).

Wa Municipality in Ghana's Upper West Region faces severe food insecurity, poverty, and child undernourishment. Despite progress through social security programs, institutional barriers hinder effective implementation (Burchi 2021). Nutritional deficiencies persist, particularly among children in rural areas within the Municipality (Ecker and van Asselt 2017). Concerns about the nutritional value of meals provided through the “school‐feeding programme” highlight the need for ongoing efforts to combat poverty, food insecurity, and nutritional deficiencies (Bigson et al. 2019).

Improving the nutritional value of food through fortification strategies such as adding egg powder to Tom Brown is crucial. However, consumer acceptance of the fortified product is also essential for success. Children are more likely to consume and benefit from a nutritionally enhanced food if it is palatable and culturally acceptable (Chakona and Shackleton 2019). Sensory evaluation, which includes taste, texture, aroma, and overall acceptability, is vital for the success of any fortification intervention (Hummel et al. 2018). Studies have shown that cultural preferences and sensory qualities significantly impact the acceptability of fortified foods (Boateng et al. 2019; Huey et al. 2022).

In spite of these, prior research on food fortification for child nutrition often focused on isolated nutrient enrichment such as iron and vitamin A or single‐ingredient interventions, neglecting the synergistic potential of combining locally available staples with underutilized protein sources such as egg powder. Thus, the wider concept of this study is to address critical gaps in multidimensional fortification, as there is limited data of evidence on composite products that simultaneously enhance macronutrients (protein, carbohydrates), critical micronutrients (iron, zinc, folate), and address energy density in a single formulation. The focus is to bridge the gap on the practical feasibility of this intervention, in the midst of scarce data on the functional properties (viscosity, solubility) and shelf life stability of egg powder‐fortified staples which are essential for scalability in low‐resource settings, as well as providing more insights into the acceptability–sustainability trade‐offs regarding the insufficient understanding of how nutrient fortification ratios such as egg powder: corn flour affect sensory preferences and compliance in study populations, especially children. The narrow objective of this study is therefore to evaluate the nutritional composition, sensory acceptability, and potential health benefits of egg powder‐fortified Tom Brown in school‐aged children, 5–12 years. The study's findings will provide valuable insights into the feasibility of this fortification strategy and its potential impact on improving child nutrition in the Wa Municipality.

## 2 Materials and Methods

The ingredients used for the formulations are roasted corn flour produced from local corn from the Wa market, whole egg powder produced commercially using the spray drying technique and with eggs of chickens from Emmppek Farms, Nigeria, Delta state, little sugar, and salt to enhance the flavor and taste.

### 2.1 Roasted Corn Flour Preparation

The *Tom Brown* used in this study was made from locally produced corn. Yellow corn was purchased from the Wa market, the bad ones carefully removed, and the remaining was roasted using dry heat in a gas‐operated oven at about 150°C–200°C. During the roasting, the corn was turned or stirred up to ensure even roasting and avoid burning. The roasting process was deemed complete when the corn turned brown in color. The roasted corn was then allowed to cool down and milled into flour (Ubbor et al. 2022).

### 2.2 Egg Powder Preparation

Egg powders find application in various food industry sectors as a flavoring and texturizing additives. They are created from whole eggs and carefully dried using various methods to increase their shelf life and produce a high‐quality final product. The egg powder utilized in this study is made commercially using the spray drying method and with chicken eggs from Emmppek Farms, Nigeria, and Delta state. The spray drying process usually involves the use of hot gas to convert liquid eggs into dry egg powder.

### 2.3 The Spray Drying Process

In order to retain the composition and consistency the liquid feed (liquid egg) was prepared by passing through pre‐processing steps such as pasteurization to ensure safety. The liquid feed was atomized into droplets to increase the surface area for efficient drying. The Nozzle Atomizers approach was used, where high‐pressure nozzles were employed to create a fine spray. In the drying chamber, hot gas (air) was introduced, where the temperature and flow rate of the hot gas were carefully controlled, and the droplets were made to come into contact with the hot gas, causing rapid evaporation of the moisture. Losing moisture from the droplets resulted in solid particles formation with shapes and sizes that are influenced by factors such as feed properties, atomization, and drying conditions. The dried particles were then separated from the drying air using a cyclone separator, and the particles were collected at the bottom. The dried egg powder was then collected and milled to obtain a uniform particle size. Airtight zip lock‐like bags were used to store the powder to prevent moisture absorption and contamination (Pirkwieser et al. 2022).

### 2.4 Mathematical Model of Spray Drying

In order to ensure accuracy and efficiency, mass and energy balance principles were used to model the spray drying process. These models took into account the heat and mass transfer between droplets and the drying medium, considering factors such as droplet size, velocity, temperature, and evaporation rate (Nagai et al. 2009). The mass balance equation accounted for the feed's mass, equating it to the sum of the dried product and the evaporated moisture, while the energy balance ensured that the energy supplied via hot gas matched the energy required for moisture evaporation. The droplet drying kinetics were analyzed using heat transfer and moisture evaporation equations. Heat transfer was modeled as *Q* = *h*·*A*·(*T*_*g*_ − *T*_*d*_) where *Q* is the heat transfer rate, *h* the heat transfer coefficient, *A* the droplet surface area, *T*_*g*_ the gas temperature, and *T*_*d*_ the droplet temperature. The moisture evaporation was however accounted for by employing d*t*/d*m* = *K*·*A*·(*P*_*s*_ −*P*_*a*_), where *m* is the moisture mass, *t* the time, *K* the mass transfer coefficient, *P*_*s*_ the saturation vapor pressure at the droplet surface, and *P*_*a*_ the vapor pressure in the surrounding air. The model thus allowed for a quantitative framework to describe the heat and mass transfer during the spray drying process (O'Sullivan et al. 2019).

### 2.5 Proximate Analysis

The proximate analysis was performed using the methods outlined by the Association of Official Analytical Chemists, AOAC, 2000 (Saputri and Putri 2024). The parameters analyzed included protein, carbohydrates, moisture, ash, and fat content. Carbohydrates were calculated by difference as described by McLoughlin et al. (2023). All laboratory analysis values were determined in triplicates and expressed as averages and compared with standard values in the literature to ensure reliability.

#### 2.5.1 Protein Determination

Protein content of the samples was determined by Kjeldahl method. About 2 g of sample was digested with H_2_SO_4_ and a catalyst, distilled with NaOH, titrated with 0.1 N HCl, and controlled with a Blank (Mæhre et al. 2018).

Calculation:

$$\%N=\frac{\left(V_{a}-V_{b}\right)\times N_{A}\times 1.401}{W}\;\text{and}\%\text{Protein}=\%N\times 6.25$$

#### 2.5.2 Nitrogen‐Free Extract (NFE)

$$\%\mathrm{NFE}=100-\left(\%\text{Moisture}+\%\mathrm{Fat}+\%\text{Crude Fiber}+\%\text{Protein}+\%\mathrm{Ash}\right)$$

#### 2.5.3 Moisture Content and Total Solids

The oven‐drying method was employed at 105°C for 5 h until constant weight was obtained (Saputri and Putri 2024).

Calculation:

$$\%\text{Moisture}=\frac{\text{Weight loss}}{\text{Initial weight}}\times 100;\%\text{Total Solid}=100-\%\text{Mixture}$$

#### 2.5.4 Ash Content

The samples were incinerated in a muffle furnace at 600°C for 2 h and the ash contents were determined as follows (Akoji et al. 2022):

$$\%\mathrm{Ash}=\frac{\mathrm{Ash}\;\text{weight}}{\text{Sample weight}}\times 100$$

#### 2.5.5 Fat Content (Soxhlet Extraction)

About 5 g of dried samples were refluxed with petroleum ether (40°C–60°C) for 6 h, and percentage fat was determined as described by Kom and Macedo (1973), and cited in (Ishak et al. 2015):

Calculation:

$$\%\mathrm{Fat}=\frac{\mathrm{Fat}\;\text{weight}}{\text{Sample weight}}\times 100$$

#### 2.5.6 Crude Fiber Determination

This was achieved through sequential digestion of samples with 1.25% H_2_SO_4_ and 1.25% NaOH, followed by filtration, drying, and ashing. Percentage crude fiber was then calculated by (Mæhre et al. 2018).

Calculation:

$$\%\text{Crude Fibre}=\frac{\text{Residue loss}}{\text{Sample weight}}\times 100$$

#### 2.5.7 Carbohydrates and Energy Content

The percentage of carbohydrate was estimated by:

$$\%\text{Carbohydrates}=100-\left(\%\text{Moisture}+\%\mathrm{Fat}+\%\text{Protein}+\%\mathrm{Ash}\right)$$

while energy was estimated via Atwater method, 4 kcal/g for protein/carbs, 9 kcal/g for fat (McLoughlin et al. 2023).

### 2.6 Mineral and Vitamin Analysis

The content of critical minerals (Iron, Zinc, Magnesium) and vitamins (A, Folate, Riboflavin) which were linked to the study variables were determined.

### 2.7 Mineral Analysis (Iron, Zinc, Magnesium)

Wet digestion technique was employed using Atomic Absorption Spectrophotometry (AAS). About 0.25 g of powdered sample was weighed into a digestion tube, and 2.5 mL conc. H_2_SO_4_, 2.5 mL conc. HNO_3_, and 1 mL perchloric acid were added to it respectively, and it was digested at 400°C until the solution turned colorless. The mixture was cooled, diluted to 50 mL with distilled water, and analyzed via AAS (El Hosry et al. 2023).

### 2.8 Folate (B9) and Riboflavin Estimation

High‐Performance Liquid Chromatography (HPLC) with fluorescence detection approached was adopted where 5 g of the sample was homogenized and added to 40 mL 0.1 M phosphate buffer (pH 7.0) and 1% ascorbic acid. The mixture was heated at 100°C for 10mins, cooled, pH adjusted to 4.5, and conjugate enzyme added It was incubated at 37°C for 2 h to allow the conversion of polyglutamates to monoglutamates. The mixture was then centrifuged at 10,000 rpm for 10 min, filtered through a 0.45 μm membrane, and 50 μL injected into HPLC for analysis. Detection was through fluorescence at Ex 280 nm/Em 360 nm, and folate content was estimated using retention time, peak area, and a standard curve. The same procedure was employed for the estimation of Riboflavin (B2) content with the use of 40 mL 0.1 M acetic acid and sodium acetate (pH 5.0) as buffer (Sosa‐Pérez et al. 2023).

General model equation for determining Folate and Riboflavin using the Calibration Curve:

The calibration curve for folate and riboflavin was prepared by plotting the peak area (or height) against the concentration of folate and riboflavin standards, respectively. The equation of the calibration curve is given by:

y=mx+b

where *y* = peak area of the standard; *m* = slope of the calibration curve; *x* = concentration of the standard; *b* = *y*‐intercept of the calibration curve.

Thus, the folate or riboflavin concentration in a sample *C*, (*C*_sample_) was determined using the peak area of sample *A*, (*A*_sample_) and the equation of the calibration curve as follows:

$$A_{\text{sample}}={\mathrm{mC}}_{\text{sample}}+b$$

and rearranging and working for sample *C*, the equation becomes:

$$N_{\text{sample}}=\frac{\left(A_{\text{sample}}-b\right)}{m}$$

for the concentration of folate or riboflavin.

### 2.9 Estimation of Vitamin A (Carotenoids and Beta Carotene)

Vitamin A (Beta carotene) was estimated by adding 5 mL acetone to 100 mg of fresh food samples, swirled gently, and 10mL petroleum ether was added to it and mixed thoroughly. The optical densities (OD) were measured at specific wavelengths using a UV spectrophotometer, and carotenoid contents were expressed in mg/g fresh sample (Saputri and Putri 2024).

$$\text{Beta carotene}=\frac{0.216\left(\mathrm{OD}.663\;\mathrm{nm}\right)-0.304\left(\mathrm{OD}.505\;\mathrm{nm}\right)+0.452\left(\mathrm{OD}.453\right)\times V}{\;d\times 1000\times W}$$

$$\text{Total carotenoids}=\frac{7.6\left(\mathrm{OD}.480\;\mathrm{nm}\right)-1.49\left(\mathrm{OD}.510\;\mathrm{nm}\right)\times V}{\;d\times 1000\times W}$$

where *V* = final volume of the extract; *d* = length of light path; *W* = fresh weight of sample; OD = optical density of a given wavelength.

### 2.10 Pasting Properties

Rapid Visco Analyzer (Perten Instrument, RVA 4500, Australia) as cited in Li et al. (2022) was used to determine the pasting properties of the samples. Weights of the samples were taken according to the moisture content of each flour sample into the flask. The paddle was placed into the canister and inserted into the instrument. The measurement cycle was initiated by depressing the motor tower of the instrument. The canister was removed upon completion of the test. All determinations were done in duplicates of three and average values recorded.

### 2.11 Shelf Life Analysis

The accelerated shelf life study model was used to assess the composite powder's shelf life, with moisture content serving as the determining factor (Keshani et al. 2015). Using an accelerated study model, the shelf life was determined by storing the product in enhanced settings with high humidity and temperatures, meant to accelerate the product's natural aging process. Moisture, flavor, texture, and other important quality parameters were tracked over time, and when they were noticeably deteriorated, the shelf life endpoint was determined. Moisture and texture were tracked or measured weekly, while flavor and color were tracked every 2 weeks, and microbial growth was tracked every 4 weeks, and deterioration of the product started at ASLT Weeks 8–12. Product stability was determined by extrapolating the data from the accelerated tests to estimate the shelf life by using the Arrhenius equation as a predictive model (Hasany et al. 2017).

### 2.12 Tom Brown Preparation, Sensory Evaluation and Acceptability Test

Three samples were prepared with different grams of egg powder and roasted corn flour (ratio) and were compared with a standard (without egg powder), making a total of four samples (Maina 2018). The ratio used for the four samples was 100:20, 100:15, 100:10, and 100:00 respectively, where the 100 g represents roasted corn flour while the varying values represent grams of egg powder (Table 1).

**TABLE 1:** Formulation formula for composite mixture of Tom Brown porridge.

Samples (g)	Sample 1	Sample 2	Sample 3	Sample 4
Ratios	400:40	400:60	400:80	400:00

*Note:* Each sample was prepared with 250 mL of water (Garcia‐Segovia et al. 2020).

From the above, the Sample 4 was used as a control. Informed consent was duly obtained from the parents/guardians on behalf of the children and was duly signed after the full details of the study were fully explained to them.

Selected primary six (6) pupils from a similar primary school were given education on how to evaluate the samples and were then made to taste the various samples and rank them using a questionnaire to describe the appearance, aroma, taste, texture/feel, and general acceptability as attributes of interest. The samples were randomly presented to the pupils, and the randomization order was generated using Compusense Cloud software. The sample size used for the evaluation was 75 pupils (Lang 2020).

From the mineral analysis conducted, 100 g of the egg powder contains 1.76 mg of iron. Then 20 g of egg powder will contain 20 × 1.76/100 = 0.35 mg iron. Thus, consuming 250 mL of fortified Tom Brown gives the child 0.35 mg × 2.5 = 0.87 mg iron from the egg powder. Also, 100 g of corn flour contains 2.61 mg of iron; hence, consuming 250 mL of the corn flour will provide 6.53 mg of iron. Thus, consuming 250 mL of the corn flour‐egg powder‐fortified Tom Brown will provide a total of 6.53 mg + 0.35 mg = 7.40 mg iron daily. Estimating a 10% loss to water during the cooking process of the Tom Brown (Moyo 2024), 10/100 × 7.40 = 0.74. Thus, total daily iron gain from Tom Brown is 7.40 mg − 0.74 mg = 6.66 mg per day consumption. The RDA for iron for children between 5 and 12 years of age used in this study is averaged at 9 mg per day (Pasricha et al. 2021). Therefore, the intervention product contributes about 74% of their RDA for iron. With regard to energy content, the conversion factor for protein, fat, and carbohydrate was used to calculate the energy contribution of the composite intervention product, yielding a total energy of 573 kcal per 250 mL serving a day, translating to about 34.7% of the average RDA of energy for children 5–12 years old. The Tom Brown was prepared daily by mixing 2000 g of roasted corn flour and 400 g of egg powder with 4800 g of added water. To estimate the daily protein contribution of the Tom Brown, total protein in corn flour and egg powder in their dry form was estimated and adjusted for after the water was used in the cooking to yield a daily protein supply of 17.34 g per day (Bierut et al. 2021).

### 2.13 Statistical Analysis

Data were analyzed using descriptive and inferential statistics to evaluate differences between intervention products (egg powder, corn flour, and composite) and sensory preferences. All experiments were conducted in triplicate, and results were expressed as mean ± standard deviation (SD). Data for pasting properties of samples are reported as descriptive statistics (direct measurements) as the focus was on characterizing physical properties rather than group comparisons. One‐way analysis of variance (ANOVA) was performed to compare mean values of carbohydrate, protein, fat, ash, moisture, minerals (iron, zinc, magnesium), and vitamins (A, folate, riboflavin) across the three samples (egg powder, corn flour, composite) as well as the hedonic scale ratings (appearance, aroma, taste, texture, acceptability) with HSD to compare means across sample ratios (100:00, 100:10, 100:15, 100:20). Tukey's HSD (post hoc test) was applied to identify significant pairwise differences between groups, with significance set at *p* < 0.05. Differences in means are denoted using superscript letters (a, b, c), where distinct letters within a column indicate statistically significant differences. Non‐linear regression models (M1: square root‐*Y* logarithmic‐*X*; M2: square root‐*Y*) were used to predict shelf life (months) based on moisture content while Relative Importance Index (RII) was calculated to rank participants' preferences.

## 3 Results

The study conducted proximate analysis on the corn flour‐egg powder mix intervention product using standard wet digestion method to determine the macronutrient content and the thermal properties. The results show that the composite product demonstrated an increase in protein content compared to the raw corn flour, with a reduction in carbohydrate content, and the differences are statistically significant as indicated in Table 2.

**TABLE 2:** Proximate composition of intervention products (% dwb).

Samples	Carbohydrate	Protein	Fat	Ash	Moisture
Egg powder	2.90 ± 0.06^a^	58.30 ± 0.13^c^	31.49 ± 0.24^c^	0.51 ± 0.64^a^	6.32 ± 0.21^b^
Corn flour	73.93 ± 0.50^b^	9.16 ± 0.54^a^	6.94 ± 0.29^a^	4.24 ± 0.42^c^	5.82 ± 0.64^a^
Composite	61.99 ± 1.04^c^	18.09 ± 0.98^b^	9.67 ± 0.09^b^	2.38 ± 0.09^b^	7.87 ± 0.06^b^

*Note:* Explanation: A standard AOAC approach was used, composite = corn flour + egg powder. Values are mean ± standard deviation on triplicate determinations. Means with different superscripts in the same column differ significantly (*p* > 0.05).

The mineral and vitamin analysis also revealed that combining corn flour with egg powder improves the mineral and vitamin contents of critical elements such as iron, zinc, magnesium, as well as vitamin A and B_9_ (Folate) which play a crucial role in human nutrition, especially among children, thus indicating the suitability of the composite product for an intervention study to improve Hb levels among children. The raw values of these elements per 100 g of the dry composite product, as well as their percentage contribution to RDA of the study participants, are shown in Table 3 while the comparisons of their means are displayed in Tables 4 and 5 respectively. With the exception of vitamin A and riboflavin, 100 g of the product contributes at least half of the average RDA of the nutrient for the referenced age group.

**TABLE 3:** Nutrient composition and % contribution to RDA composite product.

Nutrient	Amount/100 g	Average RDA for children 5–12 years	(%) Contribution to RDA
Composite minerals (mg)			
Iron (Fe)	4.69	8 mg/d	52.1
Zinc (Zn)	3.91	7 mg/d	55.8
Magnesium (Mg)	94.97	185 mg/d	50.8
Vitamins (μg)			
Vitamin A	158.7	500 mcg/d	31.7
Folate	141.1	250 mcg/d	56.4
Riboflavin	313.6 (0.31 mg)	0.7 mg/g	41.9

*Source:* Institute of Medicine (IOM), RDAs are average values for the age group.

**TABLE 4:** Mineral content of intervention products (mg/100 g).

Samples	Iron	Zinc	Magnesium
Egg powder	17.61 ± 0.83^a^	14.10 ± 0.02^c^	103.06 ± 1.32^a^
Corn flour	48.34 ± 1.03^c^	8.12 ± 0.23^a^	199.67 ± 6.24^b^
Composite	29.46 ± 2.95^b^	10.65 ± 0.33^b^	113.73 ± 2.02^a^

*Note:* Atomic Absorption Spectrophotometry (AAS) was used. Values are mean ± standard deviation on triplicate determinations. Means with different superscripts in the same column differ significantly (*p* > 0.05).

**TABLE 5:** Vitamin content of intervention products (μg/100 g).

Samples	Vitamin A	Folate	Riboflavin
Egg powder	0.06 ± 0.01^a^	91.15 ± 4.03^b^	313.20 ± 1.20^b^
Corn flour	117.85 ± 0.49^b^	27.70 ± 0.85^a^	0.75 ± 0.07^a^
Composite	154.40 ± 49.07^b^	122.70 ± 0.85^c^	313.85 ± 0.35^b^

*Note:* Explanation: A standard AOAC approach was used, composite = corn flour + egg powder. Values are mean ± standard deviation on triplicate determinations on a dry weight basis. Means with different superscripts in the same column differ significantly (*p* > 0.05).

The composite also showed significant improvements in the vitamins content compared to the corn flour alone, as shown in Table 5.

Some pasting properties and functional attributes such as viscosity, peak time, bulk density, solubility, and pasting temperature of the food product for the intervention were also investigated, and the results, as shown in Table 6, show that the composite has very good viscosity.

**TABLE 6:** Pasting properties and functional attributes of roasted corn flour‐egg powder mix.

	Peak viscosity	Breakdown viscosity	Final viscosity	Peak time	Setback	Bulk density (g/mL)	Solubility (%)	Pasting temperature
Corn flour	175	20	225	6.3	75	0.6	21	72.5
Composite	168	20.5	214	7:00	66.5	0.65	23	73.2

*Note:* Temperature is in °C, time is in minutes, viscosity in RVU, solubility in percentages, Bulk density in g/mL.

The results in Table 7 represent the shelf life of the two (2) powders used in this study as were determined using the accelerated shelf life study model with moisture content as the determinant. The findings as shown in the table did indicate that both the egg powder‐roasted corn flour mix and roasted corn flour have a storage life span of 12 months, respectively.

**TABLE 7:** Shelf life of egg powder‐roasted corn flour mix and roasted corn flour.

Sample	Statistical model	Shelf life
Egg powder‐roasted corn flour mix	M1: $\;Y={\left(2.82131-2.85042*\ln\left(X\right)\right)}^{2}$	12 months
Roasted corn flour	M2: $Y={\left(2.69926-0.930382*X\right)}^{2}$	12 months

*Note:* M1: Square root‐*Y* logarithmic‐*X* model, M2: Square root‐*Y* model, *Y*: Months, *X*: Moisture.

Similarly, the findings of the sensory evaluation and acceptability test for the egg powder‐roasted corn powder mix Tom Brown indicate that the sample with a 100:20 ratio formulation was the most preferred sample by the participants. The results also revealed that the addition of more egg powder results in an increase protein content and a decrease in carbohydrate levels, whereas an increase in egg powder addition generally results in improved sensory characteristics such as taste, aroma, appearance, as well as general acceptability. These changes are statistically significant, Table 8.

**TABLE 8:** Sensory characteristics and acceptability of egg powder mixed Tom Brown.

Attribute					
Sample	Appearance	Aroma/Flavor	Taste	Texture/Feel	General acceptability
100:00	4.09 ± 0.66^d^	4.36 ± 0.65^d^	4.32 ± 0.62^d^	4.35 ± 0.58^d^	4.51 ± 0.50^d^
100:10	3.64 ± 0.84^c^	3.65 ± 0.73^c^	3.48 ± 0.79^c^	3.64 ± 0.78^c^	3.71 ± 0.71^c^
100:15	2.52 ± 0.74^b^	2.39 ± 0.84^b^	1.99 ± 0.73^b^	2.32 ± 0.77^b^	3.20 ± 0.87^b^
100:20	1.56 ± 0.58^a^	1.36 ± 0.85^a^	1.23 ± 0.44^c^	1.36 ± 0.48^a^	1.29 ± 0.46^a^

*Note:* Sensory evaluation was conducted using a 5‐point hedonic scale. Sample ratios (100:00, 100:10, 100:15, 100:20) represent the proportions of egg powder to Tom Brown. Values are presented as mean ± standard deviation. Means with different superscripts in the same column differ significantly (*p* > 0.05).

Also, the Relative Importance Index analysis conducted further revealed that the sample with the highest egg powder concentration was best accepted by the study participants and the results are indicated in Table 9.

**TABLE 9:** Ranking of participants' best sample selection.

Sample	RII (%)	Rank
Sample1 100:10	71.73333	4th
Sample2 100:15	45.6	2nd
Sample3 100:20	26.93333	1st
Sample4 100:00	85.86667	3rd

Abbreviation: RII = relative importance index, samples are in grams.

## 4 Discussion

The study sought to evaluate the functional properties (viscosity, solubility), acceptability, and shelf life stability of egg powder‐fortified staples and provide insight into how egg powder: corn flour formulation affects sensory preferences and compliance in study populations. The proximate analysis of egg powder, corn flour, and their composite reflects varied nutritional compositions suitable to address dietary needs. Egg powder is exceptionally rich in protein (58.30%) and fat (31.49%), but contains low carbohydrates (2.90%), thus making it an ideal protein supplement (Godswill 2019). The composite exhibits a balanced macronutrient profile, with protein content increasing to 18.09%, carbohydrates reducing to 61.99%, while fat remains moderate at 9.67% upon the addition of the egg powder. Adding egg powder to the corn flour significantly enhanced the protein content of the Tom Brown, making available some17.34 g of protein per daily consumption of 250 mL of Tom Brown, representing about 65.4% of average RDA for this age group. This enhancement aligns with WHO guidelines emphasizing protein adequacy and reduced refined carbohydrates in child nutrition. The World Health Organization emphasizes 1300–2000 kcal/day for children aged 5–12 years, depending on growth spurts and local dietary patterns. The total energy contribution of the composite intervention product, as determined in this study, is 573 kcal/250 mL serving a day, which is about 34.7% of RDA, thus contributing significantly to their daily energy needs. The composite's moisture content (7.87%) and ash levels (2.38%) reflect a stable, dry product suitable for long‐term storage (Morton et al. 2018; Margeta et al. 2019). The 573 kcal/250 mL serving a day fulfilling 34.7% of the daily energy requirement makes it a practical solution for energy and protein gaps in school feeding programs (Cheng et al. 2018; WHO 2021). Fat is essential for energy and absorption of fat‐soluble vitamins, again highlighting the composite's suitability for improving dietary energy density (Wulandari and Arief 2022).

The superiority of the composite in terms of its mineral contents is demonstrated by the mineral analysis, which shows 29.46 mg/100 g iron, 10.65 mg/100 g zinc, and 113.73 mg/100 g magnesium. For children 8 to 12, one serving of the Tom Brown supplies approximately 74% of the recommended daily allowance (RDA) for iron (9 mg/day), thus contributing significantly to addressing the risk of anemia that is common in environments with inadequate resources (Gupta and Wong 2017). The greater iron (2.95 mg/100 g) and zinc (8.12 mg/100 g) content of corn flour alone highlights its vital role as a staple‐based intervention (Filho et al. 2020). The mineral composition of the maize flour aligns with earlier studies that emphasize the importance of fortified grains in global nutrition, focusing on the flour's function in cellular processes and energy metabolism (Qamar et al. 2017). The composite's combined heme (egg) and non‐heme (corn) iron sources enhance bioavailability, particularly when consumed with vitamin C (Skibsted 2016), thus making this formulation a better option to improve Hb and iron levels in children. The higher levels of iron and zinc are particularly significant for populations at risk of anemia and immune deficiencies (Kruger et al. 2015).

Besides, the composite has been shown to contain a substantial amount of magnesium, which is crucial for muscle and nerve function, thus supporting its suitability for individuals such as athletes, young children, and those with high physical activity levels and increased mineral requirements (Gupta and Wong 2017). The varied mineral content across these products suggests that their combined use will address a wide range of dietary needs, improve Hb levels among children, and promote overall health and well‐being.

The vitamin analysis further revealed that the composite product obtained enhanced levels of vitamin A, which supports immune and visual health, riboflavin, which aids energy metabolism, and folate that promotes neural development and cognitive function (Smith et al. 2023). Besides, folate has been discovered to play a major function in promoting retentive memory and academic performance among school children (Dring et al. 2022). These findings align with studies advocating fortified staples to combat micronutrient deficiencies (Imdad et al. 2022). The results also suggest that a careful combination of egg powder and corn flour results in a composite product with enhanced nutritional value, particularly with significant improvements in vitamin A, folate, and riboflavin levels, which will be ideal for interventions to improve Hb levels among school‐aged children with symptoms of mild anemia.

The composite's pasting qualities and other functional attributes confirm its appropriateness for intervention studies. Protein–starch interactions, which reduce starch swelling and enhance digestibility, are the cause of the decreased peak viscosity (168 RVU compared to 175 RVU in maize flour; Tarahi et al. 2022). Additionally, the composite flour showed a reduced setback, suggesting a decrease in retrogradation tendencies. This is probably because the amylose chain alignment may have been disturbed by a potential interaction between the egg powder's and the maize flour's carbohydrate content (Mejía Terán and Blanco‐Lizarazo 2021). Although retrogradation can affect texture and shelf life, these qualities find applications in the food sector because they are used in baked goods and ready‐to‐eat meals. On the other hand, the higher pasting temperature (Table 6) ensures thermal stability for porridge and baked goods, while lower retrogradation minimizes texture deterioration during storage (Rittenauer et al. 2017). A bulk density of 0.65 g/mL supports nutrient‐dense formulations for extruded snacks or infant foods (Chanadang and Chambers 2019). Both the composite and corn flour remained stable for 12 months with different degradation models (Liu et al. 2020). This shelf stability ensures feasibility of the product for large‐scale distribution in intervention programs.

The findings of the sensory evaluation revealed that the ratio of egg powder to corn flour significantly impacted sensory attributes. While aroma scores initially dropped with a 15 g egg powder ratio, they increased at the highest ratio (20 g), revealing complex interactions between the components (Maina 2018). With increasing egg powder concentrations, taste and texture gradually improved, reaching their peak in the sample with a ratio of 100:20. Lower percentages in the RII test indicated higher preference because the weighting scale used in this study was 1 = highest, 5 = lowest. The 100:20 ratio excelled in aroma, flavor, and acceptability, aligning with consumer trends favoring balanced fortified cereals (Olatunde et al. 2021). These findings support previous research which confirmed that egg powder improves texture and flavor in fortified foods, with higher concentrations producing superior sensory attributes (Saint‐Eve et al. 2019). Similar trends were observed in composite formulations with alternative proteins (Mohd Fauzi et al. 2022). The high acceptability of the 100:20 blend, along with its protein and micronutrient density, makes it a viable intervention to improve hemoglobin levels and cognitive outcomes in children, consistent with the positive public health effects reported on fortified foods.

### 4.1 Implications for Policymakers

Policymakers may incorporate the composite product into school meals or feeding programs, maternity health programs, or subsidized food baskets to provide the dual health benefits of satisfying energy needs through its carbohydrate content and addressing anemia through iron fortification. Purchasing grain and eggs from local farmers, forming public–private partnerships, and providing subsidies could help lower costs and increase community acceptance and patronage of the composite product to ensure its sustainability. Companies that are in the food business can create ready‐to‐mix versions of this product for caretakers that highlight nutrient density and convenience on labeling in order to attract health‐conscious consumers. Finally, by guaranteeing adherence to WHO micronutrient standards, authorities should set up quality control procedures for shelf‐stable fortified foods like this composite.

## 5 Conclusion

This study illustrated that the corn flour‐egg powder composite is a nutrient‐dense, scientifically validated solution to addressing micronutrient deficiencies and protein‐energy malnutrition in children. The balanced macronutrient profile of the composite is in line with WHO guidelines, contributing about 34.7% of daily energy needs (RDA) for children aged 5–12 years. Its high iron, zinc, and magnesium contents, coupled with enhanced bioavailability from heme and non‐heme iron synergy, make it suitable for addressing anemia risks, which are common in resource‐limited settings. The Tom Brown also showed a significant amount of vitamin A, riboflavin, and folate, which are critical requirements for immune function, cognitive development, and academic performance, thereby falling in line with global fortification strategies. Functionally, the composite's reduced peak viscosity and thermal stability (pasting temperature) enhance its digestibility and suitability for porridge, baked goods, and extruded snacks, while its 12‐month shelf life presents it as an ideal product for large‐scale distribution.

Sensory evaluations confirmed the 100:20 corn‐to‐egg powder ratio as optimal, with superior taste, texture, and acceptability, reflecting consumer preferences for balanced fortified cereals. However, further research is required to conduct field trials to assess real‐world vitamin retention during cooking, such as folate loss in boiling porridge, as well as testing bioavailability of iron by validating iron absorption rates on a larger scale in human studies with vitamin C consumption. Also, further studies could explore alternative fortification ratios and assess long‐term health outcomes in children consuming egg powder‐fortified Tom Brown. Overall, by bridging scientific innovation with practical application, this composite offers a scalable, sustainable strategy to improve child nutrition, especially among resource‐disadvantaged populations.

## Author Contributions

**Joe Dare Nyefene:** conceptualization (equal), data curation (supporting), formal analysis (lead), investigation (equal), methodology (equal), project administration (lead), resources (lead), visualization (equal), writing – original draft (lead), writing – review and editing (equal). **Felix Mills‐Robertson:** conceptualization (lead), methodology (lead), project administration (equal), resources (equal), supervision (lead), writing – review and editing (equal). **Isaac Amoah:** conceptualization (equal), methodology (supporting), supervision (equal), validation (equal), visualization (equal), writing – review and editing (equal). **Charles Apprey:** methodology (equal), resources (supporting), software (supporting), supervision (equal), writing – review and editing (equal). **Christopher Edeh:** conceptualization (equal), resources (equal), validation (supporting), visualization (supporting), writing – review and editing (equal).

## Ethics Statement

The study was approved by the Committee on Human Research, Publication and Ethics at the Kwame Nkrumah University of Science and Technology, Ghana (CHRPE/AP/998/23).

## Consent

Consent and assent of guardians and participants was duly obtained for recruitment. The confidentiality and privacy of participants were ensured throughout the study.

## Conflicts of Interest

Mr. J.D.N., the lead researcher in this work, received support by way of supply of whole egg powder from Emmppek Farms Ltd., Nigeria, Delta state. Aside from that, the authors declare no conflicts of interest.

## Data Availability

The dataset used/analyzed that supports the findings of this study is available upon reasonable request from the corresponding author.
